# *Pin-Align*: A New Dynamic Programming Approach to Align Protein-Protein Interaction Networks

**DOI:** 10.1155/2014/393908

**Published:** 2014-11-10

**Authors:** Farid Amir-Ghiasvand, Abbas Nowzari-Dalini, Vida Momenzadeh

**Affiliations:** Department of Computer Science, School of Mathematics, Statistics, and Computer Science, College of Science, University of Tehran, Tehran 1417614411, Iran

## Abstract

To date, few tools for aligning protein-protein interaction networks have been suggested. These tools typically find conserved interaction patterns using various local or global alignment algorithms. However, the improvement of the speed, scalability, simplification, and accuracy of network alignment tools is still the target of new researches. In this paper, we introduce *Pin-Align*, a new tool for local alignment of protein-protein interaction networks. *Pin-Align* accuracy is tested on protein interaction networks from IntAct, DIP, and the Stanford Network Database and the results are compared with other well-known algorithms. It is shown that *Pin-Align* has higher sensitivity and specificity in terms of KEGG Ortholog groups.

## 1. Introduction

In the last decade, high throughput techniques in experimental biology produced a large amount of biological data which usually can be modeled by large networks such as metabolic networks, gene regulatory networks, and protein-protein interaction (PPI) networks [1]. Analogous to biological sequence comparison [2–4], comparing large biological networks can improve our biological understanding of them. Comparison of PPI networks is a well-studied area due to the important roles of PPI networks. The main idea behind comparison of PPI networks is to find evolutionary conserved interaction modules which describes functional relevance. Exact comparison of large PPI networks is a NP-hard problem and there is no polynomial time algorithm for it. The NP-hardness of this problem is based on the fact that this problem can be reduced to the subgraph isomorphism problem. In this way, the exact comparison of PPI networks is computationally infeasible, and PPI networks comparison is often addressed by heuristic methods [5–7].

Several algorithms have been proposed for biological network alignment. One of the first proposed network alignment algorithms is* Path-Blast* [8]. This algorithm finds high-score conserved pathways. After that, Sharan et al. designed* Network-Blast* [9] in order to identify conserved protein complexes in multiple species. Koyuturk et al. devised an evolution-based scoring scheme to detect conserved clusters, called* MaWISh* (maximum weight induced subgraph) [10]. Flannick et al. proposed* Graemlin* as the first method to detect conserved subnetworks of arbitrary structures with progressive alignment method [11]. All these mentioned algorithms are called local alignment algorithms owing to the fact that they started to find subnetworks such as pathways and protein complexes and expanded their search results to obtain the feasible alignment. In local network alignment, each protein may be mapped to several proteins. On the other hand, global network alignment algorithm is defined to detect the best overall mapping across all parts of the input networks.* IsoRank* is the first global network alignment algorithm aiming to maximize the overall match between two networks [12]. Flannick et al. extended* Graemlin* to global network alignment by using a training set of known network alignments to learn parameters for the scoring function. This novel algorithm is called* Graemlin 2.0* and is claimed to have linear time complexity with the number of PPIs [13]. The next version of* IsoRank*,* IsoRankN* [14], uses* Nibble-Page-Rank* algorithm [15] to align input networks locally and globally. Tian and Samatova introduced a connected-components based algorithm, called* HopeMap*, for pairwise network alignment with the focus on fast identification of maximal conserved patterns [16]. After that,* GRAAL* [6] and* H-GRAAL* [1] are presented as the global network alignment algorithms based on seed-extend approaches and Hungarian algorithm, respectively. Both of* GRAAL* and* H-GRAAL* algorithms are capable of aligning any kind of network owing to the fact that they are specifically designed based on network structure and do not use sequence similarity data. Observing that the size of true orthologs across species is small comparing to the total number of proteins in all species, Tian and Samatova presented a different approach based on a precompiled list of homologs identified by KO terms [17].

In this paper,* Pin-Align* (protein interaction network alignment) algorithm is introduced as a novel local network alignment algorithm for aligning two protein-protein interaction networks. In* Pin-Align*, Hub Clustering and hierarchical clustering algorithms are applied in the heuristic and dynamic programming phases in order to reduce time and space complexity of the problem. Two different types of scoring,* Node-Scoring* and* Structural-Scoring*, are used to find the best local alignments.* Node-Scoring* is simply based on BLAST bit score [18] and* Structural-Scoring* is based on the importance of* Bridges* (*Bridges* are considerable edges, which take part in most of the paths and consequently conserved paths in the networks) in biological networks.

The* Pin-Align* algorithm is performed on the protein interaction networks of* Escherichia coli (E. coli), Salmonella typhimurium (S. typhimurium), Caulobacter crescentus (C. crescentus), human, mouse, yeast, and Drosophila melanogaste (fly)* extracted from IntAct [19], DIP [20], and Stanford Network database (SNDB) [21]. The obtained results are compared with other well-known local alignment algorithms NetworkBLAST [9], MaWISh [10], Graemlin [11], and Graemlin 2.0 [13], in a way that Graemlin 2.0 compares its results, because it is the only method that compares results of all local alignment tools against KEGG Ortholog (KO) groups [22].

## 2. Materials and Methods

**Figure 1:**
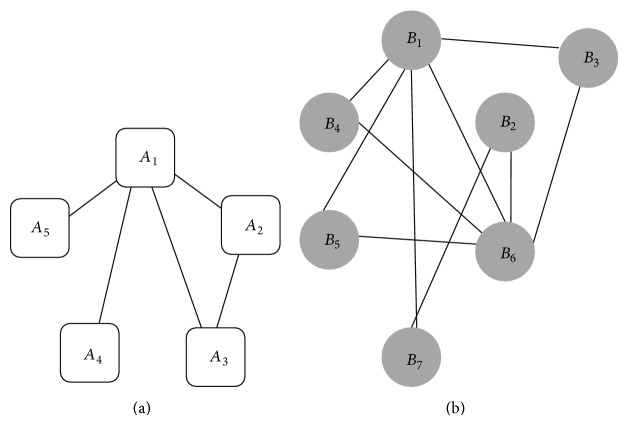
Two sample networks *A* and *B*.

Protein-protein interaction (PPI) network is defined as the set of relationships among proteins. Here, a PPI network is modeled by an undirected and weighted graph *G* = (*V*, *E*), where nodes correspond to proteins and each weighted edge specifies the probability that two proteins interact. For two graphs *G*_1_ = (*V*_1_, *E*_1_) and *G*_2_ = (*V*_2_, *E*_2_) (such as two sample networks *A* and *B* in Figure 1), the goal of network alignment is to identify possible mappings which map vertices of *G*_1_ into vertices of *G*_2_.

In addition, for each mapping, the corresponding set of conserved edges is also identified. Mappings may be partial; that is, they do not need to be defined for all the nodes of the graphs. Each mapping implies a common subgraph between the two graphs. When protein *a*_1_ from graph *G*_1_ is mapped to protein *a*_2_ from graph *G*_2_, then *a*_1_ and *a*_2_ refer to the same node in the common subgraph; the edges in the common subgraph correspond to the conserved edges [23].

**Figure 2:**
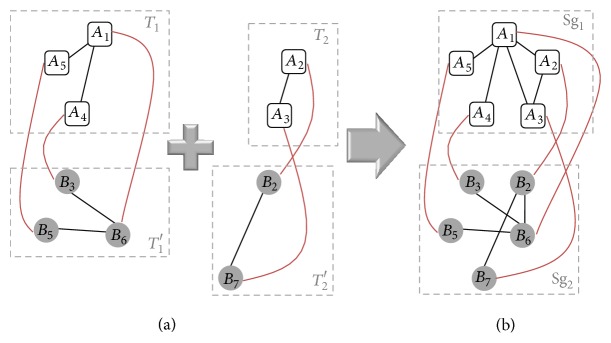
(a) Two local alignments of networks *A* and *B* of Figure 1. (b) Result of merging them.

*Pin-Align* algorithm is a local network alignment algorithm, mainly designed based on the dynamic programming approach. To explain* Pin-Align* in more detail, first a formal definition of local network alignment is presented. Given two graphs *G*_1_(*V*_1_, *E*_1_) and *G*_2_(*V*_2_, *E*_2_), local alignment LA_*G*_1_,*G*_2__(Sg_1_, Sg_2_, *M*) is a triplet where Sg_1_ and Sg_2_ are the subgraphs of *G*_1_ and *G*_2_, respectively, and *M* is defined as the mapping which aligns vertices of Sg_1_ into vertices of Sg_2_. Two sample local alignments of networks *A* and *B* of Figure 1 are shown in Figure 2(a).

Following, the best local network alignment (BLA) is defined as a function to obtain the best local alignment for each subgraph of *G*_1_. The best local alignment of Sg_1_ as a subgraph of *G*_1_ with a subgraph of *G*_2_ is shown by BLA_*G*_1_,*G*_2__(Sg_1_), which represents a local alignment with the highest score among all possible alignments between Sg_1_ and all possible subgraphs of *G*_2_ that can be mapped to Sg_1_.

Now, a naive dynamic programming algorithm for network alignment is demonstrated in the next subsection, and later* Pin-Align* algorithm is presented.

### 2.1. Dynamic Programming Algorithm to Align PPI Networks

In alignment of two weighted graphs *G*_1_ and *G*_2_ through dynamic programming technique, we generate all LAs of size one (subgraphs with a single vertex) in the first step. For obtaining LAs of larger subgraphs of *G*_1_, we merge all possible pairs of LA of smaller subgraphs. In other words, for each subgraph of *G*_1_ such as Sg_1_, there are several pairs of distinct subgraphs (subgraphs with no common vertices) such as (*T*_1_, *T*_2_) where Sg_1_ = *T*_1_ ∪ *T*_2_. Figure 2 demonstrated the merge process of two sample local alignments of networks *A* and *B* of Figure 1. We produce all possible local alignments for Sg_1_ such as LA_*G*_1_,*G*_2__(Sg_1_, Sg_2_, *M*) by merging every possible local alignments of *T*_1_ such as LA_*G*_1_,*G*_2__(*T*_1_, *T*_1_′, *M*′) with every possible local alignment of *T*_2_ such as LA_*G*_1_,*G*_2__(*T*_2_, *T*_2_′, *M*′′). This process is done iteratively, and finally all local alignments of *G*_1_ including BLA_*G*_1_,*G*_2__(*G*_1_) are obtained.

Inspired by dynamic programming technique, we propose* Simple-Align* algorithm in Algorithm 1. This algorithm can find all BLAs; however by considering time and memory complexity, this algorithm is not feasible. To overcome this, we use several heuristics to reduce memory and time complexity and propose a new local alignment algorithm,* Pin-Align*, in next section.

### 2.2. *Pin-Align* Algorithm

As explained,* Pin-Align* uses dynamic programming approach to solve the local network alignment problem and overcome the deficiencies of dynamic programming such as time and space complexity by applying heuristic approach. The steps of the* Pin-Align* algorithm are summarized as follows.

1. Partition the input graph *G*_1_ into smaller clusters. These clusters are dense subgraphs of *G*_1_. The partitioning is done by using a new clustering algorithm named Hub Clustering. Clusters obtained in this step are named* Hub-Clusters*.
2. For each *Hub*-*Cluster*_*i*_,
  (a) create local alignments of size one with BLAST bit score greater than 0, between vertices of *Hub*-*Cluster*_*i*_ and vertices of *G*_2_; local alignments obtained by this approach are named* Candidates* (*C* = LA_*G*_1_,*G*_2__(Sg_1_, Sg_2_, *M*) such that Sg_1_ is a subgraph of *Hub*-*Cluster*_*i*_ with size one),
  (b) collect all* Candidates* of subgraph Sg_1_ of *G*_1_ (such as *C* = LA_*G*_1_,*G*_2__(Sg_1_, Sg_2_, *M*)) in a new set called* Candidate-Collection* and each* Candidate-Collection* is represented by its subgraph of *G*_1_ such as CC(Sg_1_);* Candidate-Collections* obtained for single nodes of *G*_1_ are used as seeds in Step 2(c),
  (c) merge small* Candidate-Collections* (seeds) to gain larger ones. Repeat this process continuously to achieve a* Candidate-Collection *CC(*Hub*-*Cluster*_*i*_) as the final result of merging process. Size of a* Candidate* or a* Candidate-Collection* is defined by the number of vertices of its subgraph of *G*_1_ and cardinality of a* Candidate-Collection* is defined as the number of its* Candidates*. In this step, each* Candidate-Collection* can be merged with several* Candidate-Collections* and create* Candidate-Collections* with larger size. For determining which* Candidate-Collections* should be merged together, a new hierarchical clustering is used as a pattern of merging.

The* Candidate-Collections* of* Hub-Clusters* are named* Final-Candidate-Collections*.

(3) Based on* Final-Candidate-Collections*, find final alignment using similarity graph. This graph is a special weighted bipartite graph where first and second set of vertices of it are shown by *V*_1_ and *V*_2_ which are the set of vertices of *G*_1_ and *G*_2_. The weight of each edge is computed based on* Final-Candidate-Collections*.

**Figure 3:**
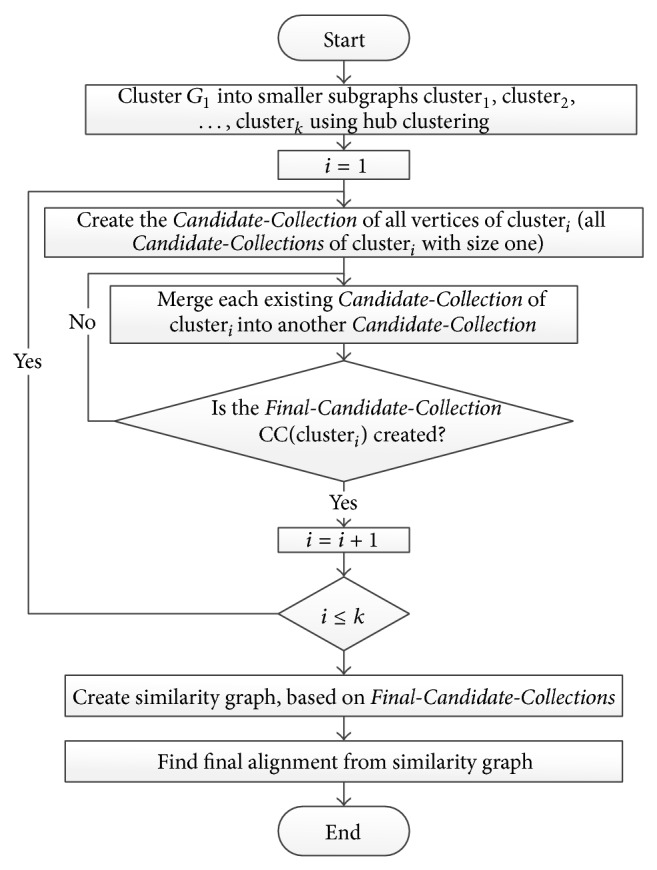
Flowchart of* Pin*-*Align* algorithm.

Flowchart of* Pin-Align* algorithm is presented in Figure 3 and its details are explained by an example on two networks *A* and *B* given in Figure 1.

In Step 1, in order to reduce the search space of the problem, input graph *G*_1_ = (*V*_1_, *E*_1_) is clustered into some dense subgraphs and Step 2 of the algorithm runs for each dense subgraph separately. Generally PPI networks are sparse graphs, but they have dense regions containing high degree vertices named hubs. These regions usually contain protein complexes; therefore conserved modules among different PPIs usually exist in these regions.

To detect dense subgraphs in the graph *G*_1_, a novel clustering algorithm, called* Hub Clustering*, is proposed. The importance of hub nodes and density of subgraphs are two major criteria for this clustering method. First, a portion of highly connected vertices are selected as hub nodes. The vertices degree of these nodes is greater than 95% of other nodes. The hub nodes are considered as the center vertices of dense regions of graph *G*_1_ and are supposed to be initial clusters. For other nodes of *G*_1_, each node is joint to the nearest cluster (the cluster that contains its nearest hub). For this aim, the length of each edge is defined as the negative logarithm of its weight (from adjacency matrix), and then Dijkstra shortest path algorithm [24] is used to find the nearest cluster. The obtained clusters are named* Hub-Clusters*.

**Figure 4:**
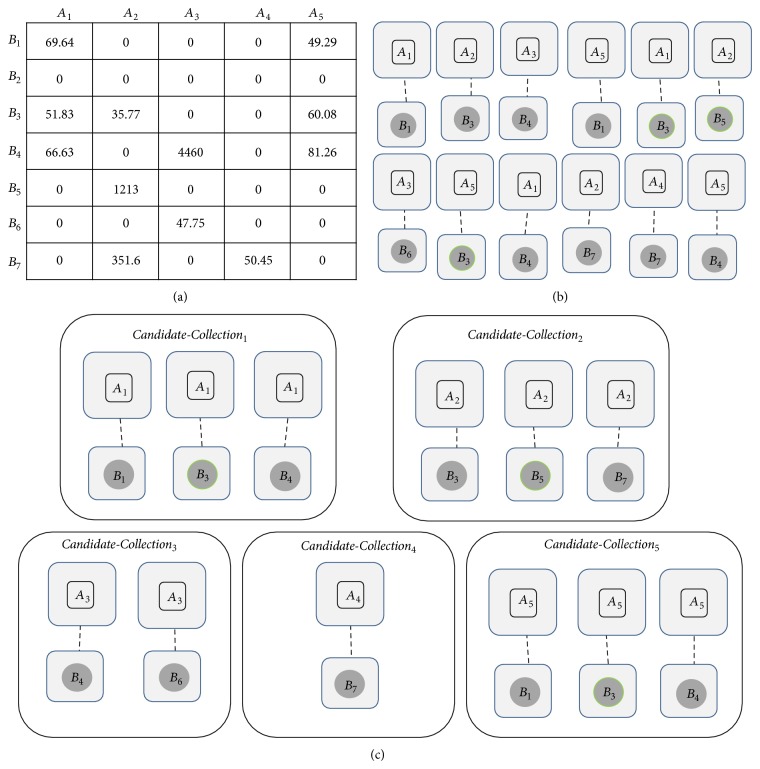
(a) BLAST bit scores for proteins of networks *A* and *B* shown in Figure 1. (b) All possible* Candidates* of size one of networks *A* and *B*. (c) All possible* Candidate-Collections* of size one for networks *A* and *B*.

In Step 2, each of* Hub-Clusters* of *G*_1_ (obtained in Step 1) is aligned to *G*_2_. As mentioned, Step 2(a) creates* Candidates* of size one based on BLAST bit score of vertices of two graphs *G*_1_ and *G*_2_ (BLAST bit score is a normalized score which is calculated by basic local alignment search tool, based on sequence similarity [18]).  Figure 4(a) shows BLAST bit scores of proteins of networks *A* and *B*.

After that, all* Candidates* of each vertex of *G*_1_ are collected into a* Candidate-Collection* with size one as a seed in Step 2(b). In other words,* Candidate-Collection* of each vertex of *G*_1_ such as Sg_1_ contains all* Candidates* for Sg_1_ from *G*_2_ which can be aligned with Sg_1_. For example,* Candidates* of networks *A* and *B* of Figure 1 are shown in Figure 4(b) and those* Candidate-Collections* are shown in Figure 4(c).

**Figure 5:**
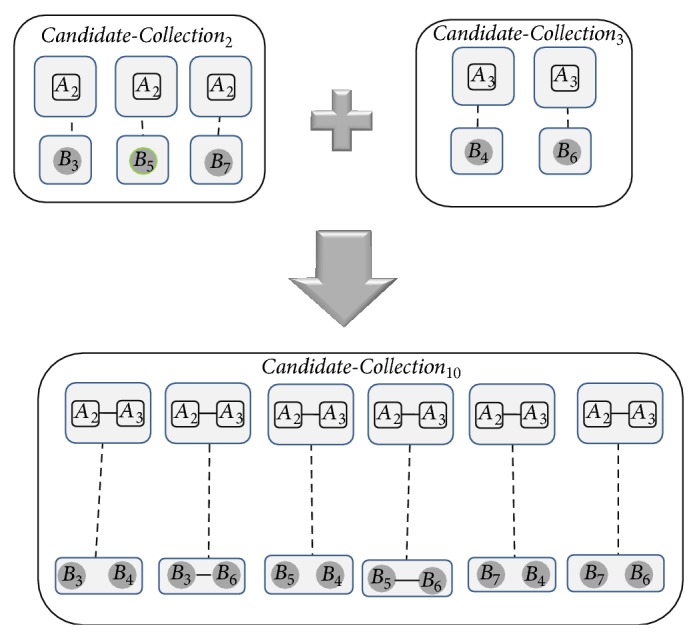
Two* Candidate-Collections* and the result of merging them.

In Step 2(c), two seeds (i.e.,* Candidate-Collections *CC(Sg_*i*_) and CC(Sg_*j*_)) are merged in order to produce a new seed (new-CC) where its size is sum of sizes of CC(Sg_*i*_) and CC(Sg_*j*_).  Figure 5 illustrates two* Candidate-Collections*, *Candidate*-*Collection*_2_ and *Candidate*-*Collection*_3_, of Figure 4(c) and the result of merging them.

The important problem in this step is discovering the feasible combining pattern to find pairs of* Candidate-Collections* where merging them generates high score* Candidate-Collections*. In order to find high score* Candidates* and avoid complex searches, combining pattern for merging* Candidate-Collections* should produce* Candidates* with more conserved edges. Hence we use hierarchical clustering as combining patterns [25]. Hierarchical clusters are generally constructed by generating a sequence of partitions where each subcluster belongs to one supercluster in its entity.

In hierarchical clustering we should determine how the distance between two clusters is computed. If *S*_*i*_ = (*V*_*i*_′, *E*_*i*_′) and *S*_*j*_ = (*V*_*j*_′, *E*_*j*_′) are subgraphs of *G*_1_ = (*V*_1_, *E*_1_), the distance between two* Candidate-Collections *CC(*S*_*i*_) and CC(*S*_*j*_) is computed, using the following formula:

(1) $$\begin{array}{c} \text{dis}\left(\text{CC}\left(S_{i}\right),\text{CC}\left(S_{j}\right)\right)=\frac{\left|V_{i}^{\prime }\right|+\left|V_{j}^{\prime }\right|}{\sum_{v\in V_{i}^{\prime }}^{}\sum_{u\in V_{j}^{\prime }}^{}\left(a_{v,u}/e_{v,u}\right)}. \end{array}$$

In this equation, *a*_*v*,*u*_ is equal to one if there is an edge between vertices *u* and *v*; otherwise it is zero, and *e*_*v*,*u*_ is the negative logarithm of probability of interaction between two proteins *v* and *u* in the graph *G*_1_. We use the hierarchical clustering obtained based on formula, as the combining pattern for merging* Candidate-Collections*. Cardinality of new* Candidate-Collections* obtained in higher levels of hierarchical clustering tree is increased exponentially as a result of merging smaller* Candidate-Collections*, so we have to save the* Candidates* with highest score and discard* Candidates* with low scores. In Step 2(c) we use a multiobjective optimization technique for finding the highest score* Candidates* [26]. The highest score* Candidates* are obtained by sorting* Candidates* based on two criteria, first,* Nodes-Scores*; next,* Structural-Scores*. Finally, the* Candidates* with the highest scores are chosen in each step. Following, we describe this scoring schema.

*Node-Score*.* Node-score* of each* Candidate C* = LA_*G*_1_,*G*_2__(Sg_1_, Sg_2_, *M*) is obtained by using the following equation:

(2) NodeScore(C) =∑i=1Sg1BLASTBitScoreSg1i,MVSg1i,M.

In this equation, *i* is an iterator on vertices of Sg_1_ and Sg_1_^*i*^ is the *i*th vertex of Sg_1_; MV(*v*, *M*) indicates a vertex of Sg_2_ mapped into *v* ∈ Sg_1_ by the mapping *M*. 

*Structural-Score*. Due to the existence of hubs and small words in biological networks, certainly the network contains bridges. Bridges are considerable edges, which take part in most of the paths (specifically conserved paths) in the networks. Because of the importance of bridges, if Sg_1_ = (*V*_1_′, *E*_1_′) is a subgraph of *G*_1_ = (*V*_1_, *E*_1_) and Sg_2_ = (*V*_2_′, *E*_2_′) is a subgraph of *G*_2_ = (*V*_2_, *E*_2_), the structural scoring function for each* Candidate C* = LA_*G*_1_,*G*_2__(Sg_1_, Sg_2_, *M*) is defined as follows:

(3) StructuralScore(C) =∑v∈V1′∑ u∈V1′fv×fuα  ×PG1(v,u)PG2MVv,M,          MVu,M1−α.

In this equation, *f*(*i*) shows the number of conserved edges at vertex *i* in the subgraph Sg_1_; *α* is a constant value between 0 and 1 and shows the importance of adjacent vertices degrees; *P*_*G*_(*x*, *y*) is the probability of interaction between protein *x* and protein *y* in the network G.

As mentioned before, to control the exponential growth of the cardinality of* Candidate-Collections* in combining process, we use a multiobjective optimization method on node score and structural score, as optimization objectives. In this way, after creating each* Candidate-Collection* among all* Candidates* obtained in that, we select only a limited number of* Candidates* (based on upon scoring functions) and discard others. Obtained* Candidate-Collections* of* Hub-Clusters* are named* Final-Candidate-Collections* which contain* Final-Candidates *(*Candidates* of* Hub-Clusters*).

After creating all* Final-Candidate-Collections*, in Step 3 of* Pin-Align* Algorithm, an* Initial Similarity Bipartite Graph *ISBG(*G*_1_, *G*_2_) = (*V*_1_, *V*_2_, *E*) is created which consists of vertices of *G*_1_ = (*V*_1_, *E*_1_) as its first part and vertices of *G*_2_ = (*V*_2_, *E*_2_) as its second part, and *E* is a set of weighted edges between *V*_1_ and *V*_2_. The weight of each edge represents the similarity between its incident vertices. Let *C*(*v*_*i*_, *u*_*j*_) be a set of all* Final-Candidates* which maps *v*_*i*_ into *u*_*j*_. In ISBG(*G*_1_, *G*_2_), the weight of an edge *e* = {*v*_*i*_, *u*_*j*_}, where *v*_*i*_ ∈ *V*_1_ and *u*_*j*_ ∈ *V*_2_, is equal to zero if |*C*(*v*_*i*_, *u*_*j*_)| = 0; otherwise this weight is defined by the following formula:

(4) Wvi,uj=NodeSimvi,uj+NeighborhoodsSimvi,uj×1|C(vi,uj)|,

where Node_Sim_(*v*_*i*_, *u*_*j*_) is the similarity between two proteins *v*_*i*_ and *u*_*j*_ and it is calculated by using the following formula:

(5) $$\begin{array}{c} \text{Nod}{\text{e}}_{\text{Sim}}\left(v_{i},u_{j}\right)=\text{BLASTBitScore}\left(v_{i},u_{j}\right)\times \left|C\left(v_{i},u_{j}\right)\right|, \end{array}$$

Neighborhoods_Sim_(*v*_*i*_, *u*_*j*_) is the similarity between conserved neighborhoods of *v*_*i*_ and *u*_*j*_ and is calculated as follows:

(6) NeighborhoodsSim(vi,uj) =∑c∈C(vi,uj) ∑(vi′,uj′)∈CNc(vi,uj)BLASTBitScore(vi′,uj′).

Let *N*_*G*_(*v*) be the set of all neighbors of *v* (*N*_*G*_(*v*) = {*u* ∈ *V*(*G*)∣*uv* ∈ *E*(*G*)}); then CN_*c*_(*v*_*i*_, *u*_*j*_) for* Candidate c* = LA_*G*_1_,*G*_2__(Sg_1_, Sg_2_, *M*) is

(7) $$\begin{array}{c} \text{C}{\text{N}}_{c}\left(v_{i},u_{j}\right)=\left\{\left(v_{i}^{\prime },\text{MV}\left(v_{i}^{\prime },M\right)\right)\;|\;v_{i}^{\prime }\in N_{G_{1}}\left(v_{i}\right)\right\}. \end{array}$$

**Figure 6:**
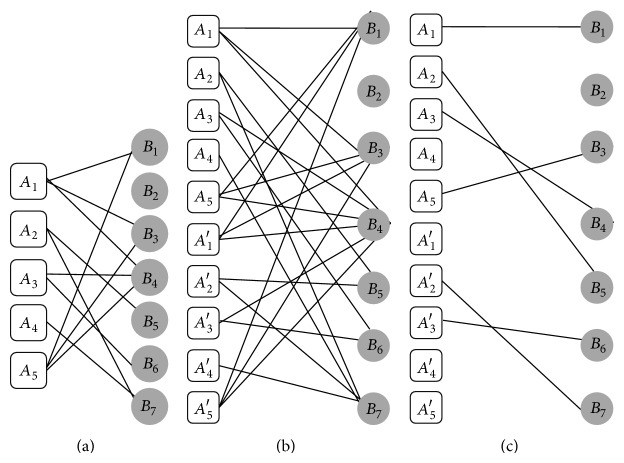
(a) The* Initial Similarity Bipartite Graph* (ISBG) between networks *A* and *B* in Figure 1 (the weights of edges are hidden for simplicity). (b) The ISBG, with duplication of first part. (c) Graph *M*_1_, obtained from first part of duplicated ISBG by performing Hungarian algorithm.

An* Initial Similarity Bipartite Graph* for networks *A* and *B* in Figure 1 is shown in Figure 6(a). After creating the* Initial Similarity Bipartite Graph* (ISBG), we can find just a single mapping for each vertex by finding maximum matching in this graph. The maximum matching can be found by applying the Hungarian algorithm (with polynomial time complexity) on the ISBG [27].

As mentioned, this approach can find just a single mapping for each vertex, although it cannot support some evolutionary functions such as duplication and diversion. To support duplication and diversion, we should consider matching of a single vertex from one part into two vertices of other parts in the ISBG. This type of matching is named* DMatch*. In other words,* DMatchs* are paths with length 2 such as *P*(*v*_*i*_′, *v*_*j*_, *v*_*k*_′) in the ISBG which aligns *v*_*j*_ from one graph into both *v*_*i*_′ and *v*_*i*_′ from another graph. Here the problem is to find a set of* Matchs* and* DMatchs* from the ISBG where sum of their weights is maximum. This problem is named* Maximum Weighted DMatching*. To decrease the size of the solution space of this problem, we decrease the size of edge set of the ISBG by deleting some edges. The above procedure is performed by Algorithms 2 and 3.

**Figure 7:**
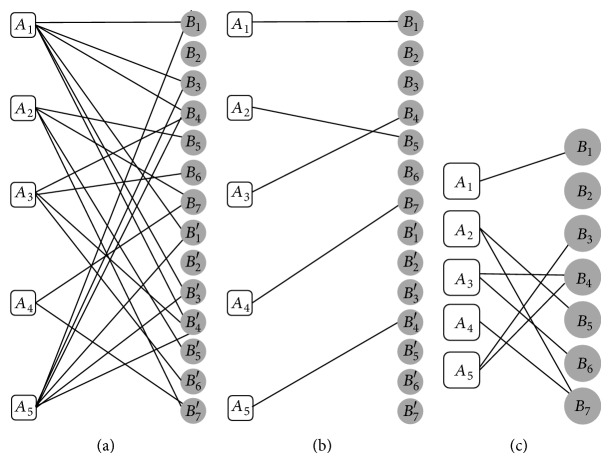
(a) The ISBG between networks *A* and *B* in Figure 1, with duplication of second part. (b) Graph *M*_2_, obtained from second part duplicated ISBG by performing Hungarian algorithm. (c) The* Final Similarity Bipartite Graph* (sim_*G*_) for networks *A* and *B*.

Algorithm 2 gives the ISBG as input and creates the* Final Similarity Bipartite Graph*. In Steps 1–4 of Algorithm 2 all vertices of one part of the ISBG are duplicated and each duplicated vertex is connected to all vertices conjunct to the main vertex with the same weight as shown in Figure 6(b); then the Hungarian algorithm is performed on this graph in Step 5 of Algorithm 2. Obtained bipartite graph is named *M*_1_. The result of these steps for ISBG of Figure 6(a) is shown in Figure 6(c). All duplicated vertices of *M*_1_ are merged together in Step 6 of Algorithm 2. In Steps 7–12 of Algorithm 2, the same process is implemented on the other part of the obtained ISBG and the matching *M*_2_ is obtained. Figures 7(a) and 7(b) illustrate these steps for ISBG of Figure 6(a). Step 13 of Algorithm 2 combines *M*_1_ and *M*_2_ to create Sim_*G*_ as the* Final Similarity Bipartite Braph* as shown in Figure 7(c).

It can be proven that* Maximum Weighted Dmatchings* of graph Sim_*G*_ are exactly similar to* Maximum Weighted Dmatchings* of ISBG. So Sim_*G*_ contains all edges of* Maximum Weighted Dmatching* and the degree of its vertices is at most 3. For this reason obviously the solution space of the* Maximum Weighted DMatching* problem on Sim_*G*_ is considerably decreased in comparison with the ISBG.

To find the final alignment using the graph Sim_*G*_, we use a greedy algorithm with polynomial time complexity as shown in Algorithm 3. Algorithm 3 gives the* Final Similarity Bipartite Graph *Sim_*G*_ (obtained by Algorithm 2) as input and matches all pairs of adjacent vertices where degree of both of them is one. For other vertices, Algorithm 3 creates all possible* Dmatchs* and selects them in a greedy manner. Algorithm 3 utilizes the following steps for all vertices of graph Sim_*G*_.

1. If the degree of the vertex *v* is one and degree of adjacent vertex is also one, these two vertices are assigned to each other.
2. If the degree of the vertex *v* is two and *v* is adjacent to *v*_*i*_′ and *v*_*j*_′, the path *P*(*v*_*i*_′, *v*, *v*_*j*_′) is created as a potential* Dmatch*.
3. If the degree of the vertex *v* is three and *v* is adjacent to *v*_*i*_′, *v*_*j*_′, and *v*_*k*_′, potential* Dmatchs P*(*v*_*i*_′, *v*, *v*_*j*_′), *P*(*v*_*i*_′, *v*, *v*_*k*_′), and *P*(*v*_*j*_′, *v*, *v*_*k*_′) are created.

The weight of a given* DMatch* can be calculated by the following formula:

(8) $$\begin{array}{c} W\left(P\left(v_{i}^{\prime },v,v_{j}^{\prime }\right)\right)=W\left(v_{i},v\right)+W\left(v,v_{j}\right). \end{array}$$

**Figure 8:**
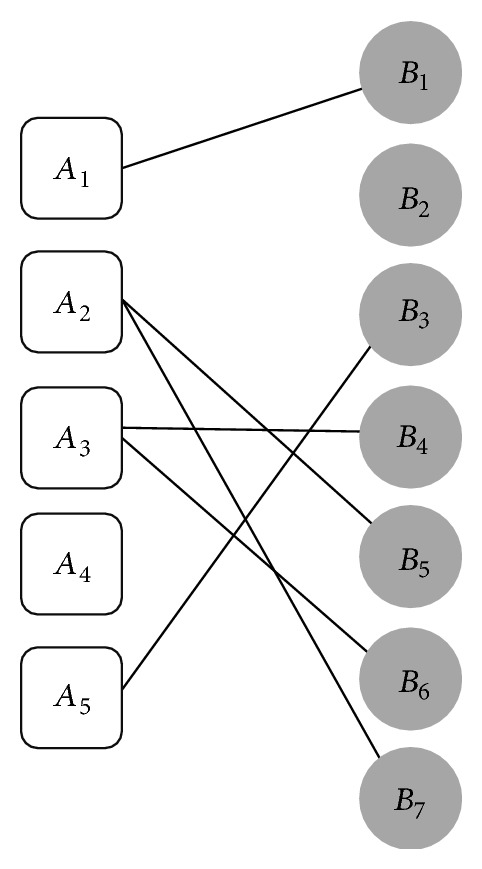
Final alignment between networks *A* and *B*.

After creating all potential* DMatchs*, we sort them based on their weights; then in a successive rounds we choose the highest weight* DMatchs P*(*v*_*i*_′, *v*, *v*_*j*_′) and delete all other edges connected to *v*_*i*_′, *v*, or *v*_*j*_′. By this way all assignments are found and finally the local alignment of two graphs is obtained in Steps 24–27 of Algorithm 3. Therefore the output of this algorithm is the final alignment between two given graphs *G*_1_ and *G*_2_. The final alignment between networks *A* and *B* in Figure 1 is shown in Figure 8.

## 3. Results

In this section, we intend to compare our algorithm to other well-known local alignment algorithms (NetworkBLAST [9], MaWISh [10], Graemlin [11], and Graemlin 2.0 [13]), in a way that Graemlin 2.0 compares its results, because it is the only method that compares results of all local alignment tools against KEGG Ortholog (KO) groups [22] on different data sets. The data sets contain the PPI networks data from SNDB [21] for the organism pairs (*E. coli*,* S. typhimurium*) and (*E. coli*,* C. crescentus*), PPI networks data from DIP [20] for the organism pair (*human, mouse*), and data from IntAct [19] database for the organism pair (*yeast, fly*). The comparison is proceeded based on specificity and sensitivity in terms of KO groups introduced in Graemlin 2.0 [13]. In short—as Graemlin 2.0 defined—to uniquely specify an alignment, the mapping should be transitive; that is, if protein *A* is aligned with proteins *B* and *C*, then protein B must also be aligned with protein *C*. Mathematically, this means that the mapping is an equivalency relation, so groups of aligned proteins are referred to as equivalence classes. An equivalent class is defined as correct if all protein members in the class are in the same KO group. To measure specificity, Graemlin 2.0 computed two metrics:

1. the fraction of equivalence classes that were correct (*C*_eq_),
2. the fraction of nodes that were in correct equivalence classes (*C*_node_),

and to measure sensitivity, it computed two metrics:

1. the total number of nodes that were in correct equivalence classes (*C*_or_),
2. the number of equivalence classes that contained 2 species (Tot).

**Figure 9:**
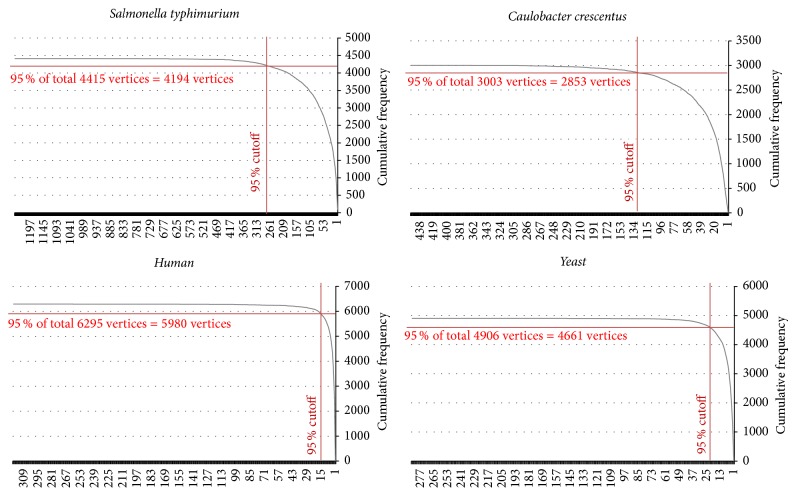
Cumulative frequency of vertices degrees in networks of* S. typhimurium, C. crescentus, human,* and* yeast*.

As we described in Step 1 of the* Pin-Align* algorithm,* Pin-Align* chooses some vertices as hubs whose degrees are greater than about 95% of total vertices. For networks* S. typhimurium, C. crescentus, human,* and* yeast*,* Pin-Align* chooses vertices as hubs in a way that their degrees are greater than 264, 126, 16, and 23, respectively. The reason why these degrees are greater than 95 percent of total vertices degrees is clear from cumulative frequency of their vertices degrees which is shown in charts of Figure 9. For example, in the PPI network of* S. typhimurium* the degrees of about 95% of vertices are less than 264, so in this network we choose vertices with degrees greater than 264 as hubs.

Table 1 shows* Pin-Align* results with different hub degrees. Column two of this table (percentage column) contains different percentages which are used for hub clustering step (Step 1).

The results of the algorithms are demonstrated in Tables 2 and 3. In these tables NB stands for NetworkBlast, MW for MaWISh, Gr for Graemlin, and Gr 2.0 for Graemlin 2.0. They show that* Pin-Align* is the most specific and sensitive aligner in comparison with other alignment tools like NetworkBlast, MaWISh, Graemlin, and Graemlin 2.0. Because Graemlin 2.0 for each of its comparisons to other local aligner removed equivalence classes of its output that did not have a node in common with output of another local aligner, we have different results of Graemlin 2.0. Graemlin 2.0 did this due to considerations of local aligners to nodes in conserved modules and global aligners to all nodes.

In Table 2, we use the PPI network data from SNDB for the organism pairs (*E. coli*,* S. typhimurium*) and (*E. coli*,* C. crescentus*). In Table 3 we use the PPI networks data from DIP and IntAct for the organism pairs (*human*,* mouse*) and (*yeast*,* fly*).

**Figure 10:**
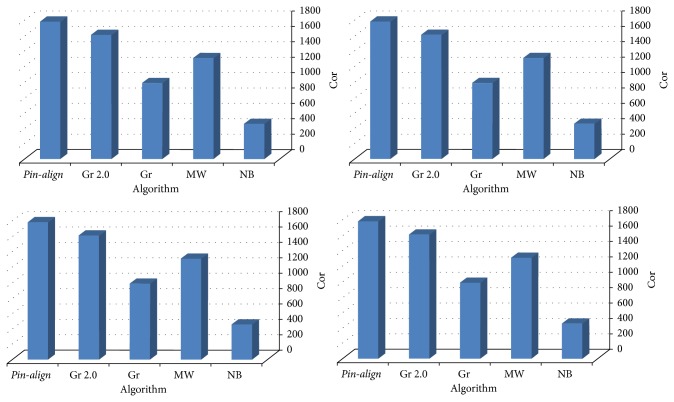
The number of correct equivalence classes that are found in alignment of organism pairs (*E. coli*,* S. typhimurium*), (*E. coli*,* C. crescentus*), (*human*,* mouse*), and (*yeast*,* fly*) by* Pin-Align*, in comparison with Graemlin 2.0, Graemlin, MaWISh, and NetworkBlast.

Figure 10 shows that* Pin-Align* also finds more correct equivalence classes than NetworkBlast, MaWISh, Graemlin, and Graemlin 2.0. By considering all the above results and comparing* Pin-Align* to the other algorithms, it is evident that* Pin-Align* is more accurate in most cases based on the *C*_eq_, *C*_node_, *C*_or_, and Tot measures.

## 4. Conclusions

In this paper, we presented* Pin-Align*, a pairwise local network alignment to improve accuracy of the alignment.* Pin-Align* algorithm is mainly designed based on the dynamic programming approach and has specificity and sensitivity comparable with existing tools such as NetworkBlast, Graemlin, Graemlin 2.0, and MaWISh. Our novel scoring system is based on bridges which are considerable edges due to the existence of hubs and small words in biological networks which certainly contain bridges. Bridges take part in most of the paths and consequently conserved paths in the networks. In future work, we plan to extend this approach to multiple network alignment.

## Figures and Tables

**Algorithm 1:**
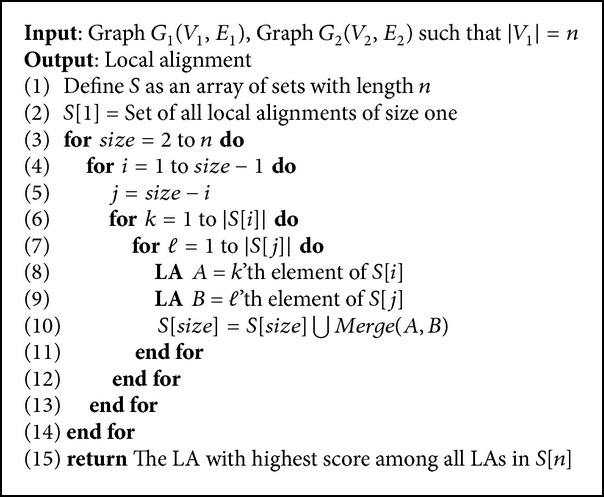
*Simple-Align* algorithm.

**Algorithm 2:**
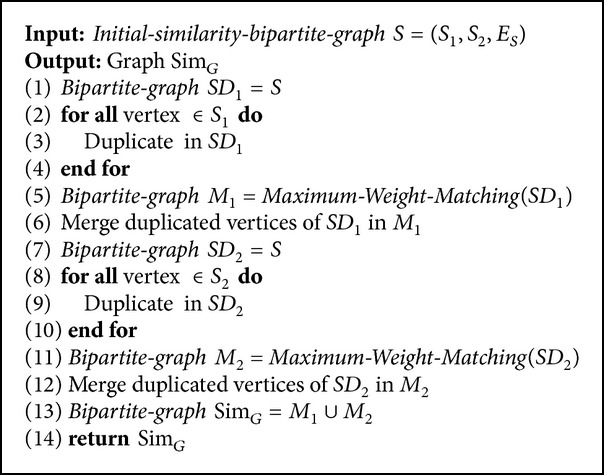
Find Sim_*G*_ Graph.

**Algorithm 3:**
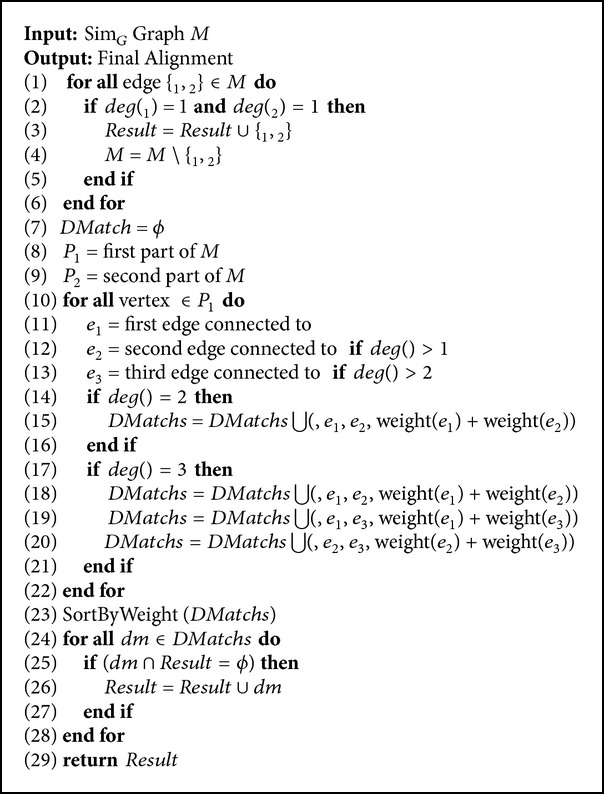
Find final alignment.

**Table 1:** Results of *Pin-Align* algorithm on four pairs of PPI networks, using different percentages in hub clustering step (Step  1).

	Percentage	*C*_eq_	*C*_node_	*C*_or_	Tot
(*E. coli*, *S. typhimurium*)	60%	0.95	0.93	1651	1779
	70%	0.95	0.94	1723	1835
	80%	0.95	0.94	1773	1890
	90%	0.95	0.94	1782	1905

(*E. coli*, *C. crescentus*)	60%	0.87	0.84	666	794
	70%	0.89	0.86	665	772
	80%	0.88	0.85	693	811
	90%	0.89	0.87	764	876

(*Human*, *mouse*)	60%	0.78	0.72	217	299
	70%	0.82	0.78	248	316
	80%	0.78	0.73	226	309
	90%	0.82	0.78	258	329

(*Yeast*, *fly*)	60%	0.87	0.85	258	303
	70%	0.89	0.87	280	323
	80%	0.91	0.88	350	396
	90%	0.91	0.89	416	466

**Table 2:** PPI networks from SNDB for the organism pairs (*E.  coli*, *S. typhimurium*) and (*E.  coli*, *C. crescentus*).

	(*E. coli*, *S. typhimurium*)				(*E. coli*, *C. crescentus*)			
	*C*_eq_	*C*_node_	*C*_or_	Tot	*C*_eq_	*C*_node_	*C*_or_	Tot
NB	0.77	0.49	457	1016	0.78	0.50	346	697
Gr2.0	0.95	0.94	627	667	0.79	0.78	447	573

MW	0.84	0.64	1309	2050	0.77	0.54	458	841
Gr2.0	0.97	0.96	1611	1678	0.77	0.76	553	727

Gr	0.80	0.77	985	1286	0.69	0.64	546	847
Gr2.0	0.96	0.95	1157	1217	0.82	0.81	608	750

**Pin-Align **	**0.95**	**0.94**	**1782**	**1905**	**0.89**	**0.87**	**764**	**876**

**Table 3:** PPI networks from DIP and IntAct for the organism pairs (*human*, *mouse*) and (*yeast*, *fly*).

	(*Human*, *mouse*)				(*Yeast*, *fly*)			
	*C*_eq_	*C*_node_	*C*_or_	Tot	*C*_eq_	*C*_node_	*C*_or_	Tot
NB	0.33	0.06	65	1010	0.39	0.14	43	306
Gr2.0	0.83	0.81	228	281	0.58	0.58	155	267

MW	0.59	0.36	87	241	0.45	0.37	10	27
Gr2.0	0.88	0.86	181	210	0.90	0.91	20	30

Gr	0.59	0.53	108	203	0.33	0.29	35	122
Gr2.0	0.86	0.84	151	179	0.61	0.61	57	93

**Pin-Align **	** 0.82**	**0.78**	**258**	**329**	**0.91**	**0.89**	**416**	**466**
